#  Exchanges of economic plants along the land silk road

**DOI:** 10.1186/s12870-022-04022-9

**Published:** 2022-12-29

**Authors:** Guangyan Wang, Qian Chen, Ya Yang, Yuanwen Duan, Yongping Yang

**Affiliations:** 1 Germplasm Bank of Wild Species, Kunming Institute of Botany, Chinese Academy of Sciences, Kunming, 650201 China; 2 Institute of Tibetan Plateau Research at Kunming, Chinese Academy of Sciences, Kunming, 650201 China

**Keywords:** Crop species, Migration route, Conflict, Consistency, The Land Silk Road

## Abstract

**Backgroud:**

The greatest contribution of the Silk Road is to communicate among different countries and nationalities, and promote two-way cultural exchanges between the East and the West. We now have clearer understanding about how material civilization and religious culture of Central Asia and West Asia spread eastward along the Land Silk Road. However, there is controversial about how crops migrate along the Land Silk Road.

**Results:**

We summarize archaeology, genetics, and genomics data to explore crop migration patterns. Of the 207 crops that were domesticated along the Land Silk Road, 19 for which genomic evidence was available were selected for discussion.

**Conclusions:**

There were conflicting lines of evidence for the domestication of Tibetan barley, mustard, lettuce, buckwheat, and chickpea. The main reasons for the conflicting results may include incomplete early knowledge, record differences in different period, sample sizes, and data analysis techniques. There was strong evidence that Tibetan barley, barley, wheat, and jujube were introduced into China before the existence of the Land Silk Road; and mustard, lettuce, buckwheat, chickpea, alfalfa, walnut, cauliflower, grape, spinach, apple, cucumber, mulberry, and pea spread to China via trade and human migration along the Land Silk Road.

**Supplementary Information:**

The online version contains supplementary material available at 10.1186/s12870-022-04022-9.

## Introduction

The Belt and Road Initiative promises to be the largest infrastructure project in human history, and its main aims are to increase regional connectivity and economic integration [1, 2]. It includes the Silk Road Economic Belt and the 21^st^ Century Maritime Silk Road, and spans 65 countries (including China) across mainland Eurasia, Africa, and Middle East. The initiative has five components: policy coordination, transport connectivity, trade facilitation, currency convertibility, and people–to–people exchange [3]. Such a large–scale project will necessarily pass through environmentally fragile regions and key biodiversity areas. The biodiversity hotspots along the route are habitats for more than 4,138 animals and 7,371 plant species [4]. Yet, 10,000 years ago, people depended mainly on foods consisting of those plants and animals [5]. Even more remarkably, great changes in human production and lifestyle took place at that time, with two of the earliest centers of domestication worldwide located in the East and the West of the Eurasian continent. Wheat and barley as well as cattle and sheep were domesticated in the Fertile Crescent, and rice and millet were domesticated in the Yangtze and Yellow River basins, respectively [6, 7]. Such transcontinental cultural interactions within the Eurasian continent promoted the formation of the ancient Silk Road, which opened up a convenient channel for exchange between Eastern and Western civilizations [8]. Zhang Qian’s visit to the Western Regions is regarded as a symbol of the opening of the Silk Road in 138 BC [9]. Two visits to the Western Regions broke the nomads’ monopoly in the Silk Road trade, allowing for the establishment of direct trade relationships among China, Central Asia, and West Asia [10]. Exchanges along the route have lasted for thousands of years and have had far–reaching effects, especially in terms of agriculture. Agricultural exchange has been, and is still, a two–way interaction. For example, the introduction and promotion of West Asian wheat and American maize in China have had a profound and extensive impact on China’s economic and social development, and the introduction of peppers from Central and South America changed people’s lifestyles in many provinces in China. Likewise, the spread of Chinese traditional agriculture to the outside world has profoundly affected the development of agricultural around the world. Therefore, the outward spread of crops originating from China has affected the pattern and appearance of agricultural production worldwide, while the introduction of crops from other regions into China has also affected crop planting structure, crop diversity, food culture, and material life in China [11].

The opening of the Silk Road linked East Asia with Central Asia [12]. East Asian flora was closely related to Central Asian flora, which was mainly reflected by the 13th type (Central Asia distribution) and its five subtypes of Flora, comprising 139 genera in six families [13]. Some of these plant species travelled between East Asia and Central Asia with the expansion of trade exchanges. For instance, rice, soybean, and mulberry spread from China to Central Asia, and cotton, sugarcane, and hyacinth spread from Central Asia to China. However, there is still much debate about the migration process of many crops because of conflicting evidence between archaeology and genetics. For example, common bean (*Phaseolus vulgaris* L.) originated from Southern Mexico and Mesoamerica according to “the Center of Origin Theory” [14]. Based on archaeological, historical, botanical, and biochemical evidence, Southern Mexico and Mesoamerica as well as South America were two independent centers of origin [15]. Phylogeographic evidence suggested two migration events: one from Mesoamerica to South America, and the other from northern America to Mesoamerica [16]. Today, genome sequencing and assembly is a useful strategy for advancing our understanding of domestication. Genome technologies include Sanger dideoxy DNA sequencing technology and next–generation sequencing (NGS) [17]. Importantly, studies on crop domestication using NGS have verified that the migration route of common bean was from Mesoamerica to the Andes [18]. In the present study, we synthesize information from archaeology, genetics, and genomics studies to explore the migration process of crops along the Land Silk Road. First, we estimate how many crops move along this route; and second, we determine whether archaeology are consistent with genetics for which genomic data is available. In some cases, different migration patterns are suggested by genetics and archaeology. We discuss the main reasons for these inconsistencies.

## Results and discussion

**Fig. 1:**
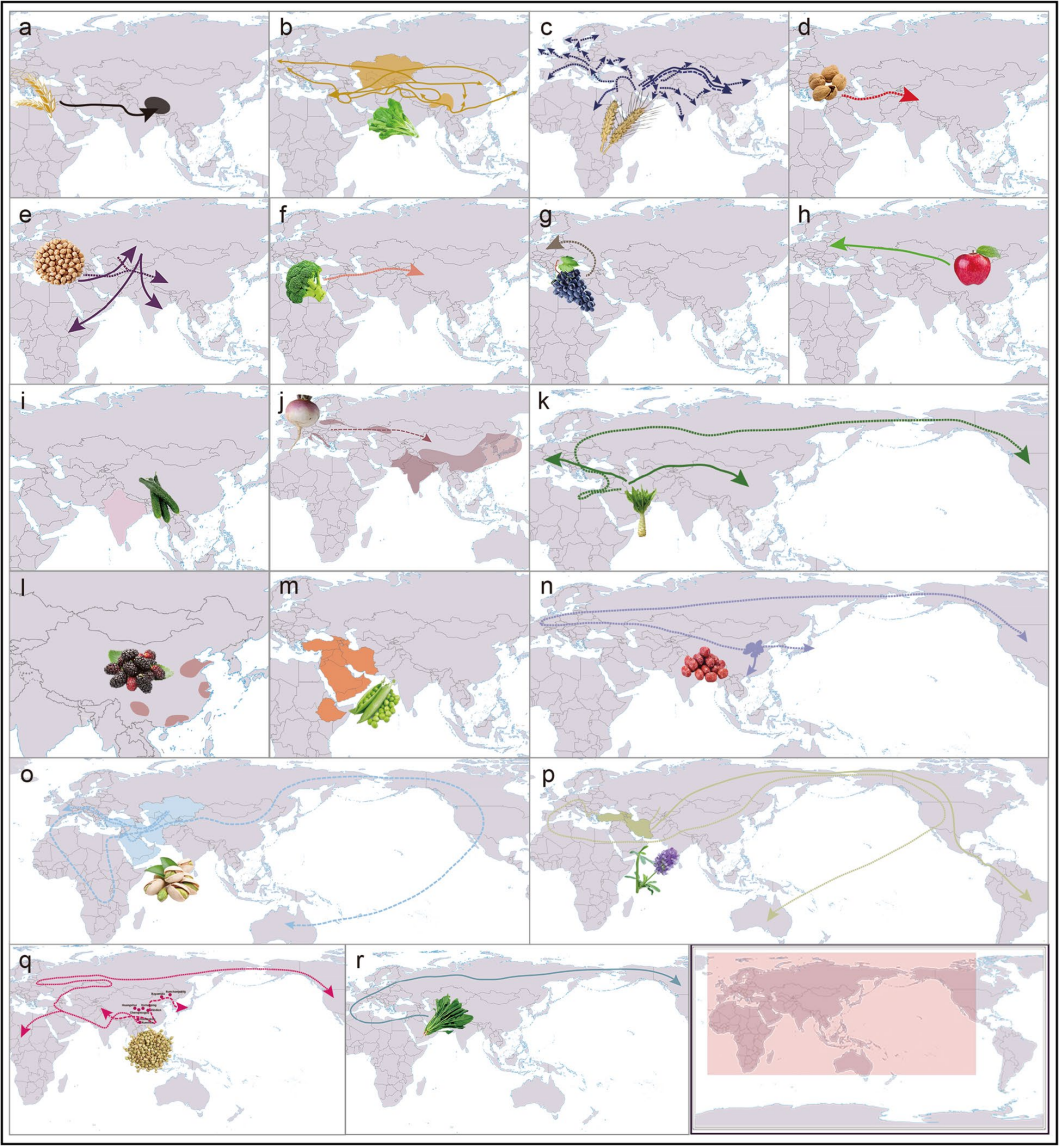
Migration routes of 19 important crops along the Land Silk Road based on different lines of evidence. a. Tibetan barley; b. Mustard; c. Barley and Wheat; d. Walnut; e. Chickpea; f. Cauliflower; g. Grape; h. Apple; i. Cucumber; j. Turnip; k. Lettuce; l. Mulberry; m. Pea; n. Jujube; o. Pistachio; p. alfalfa; q. Buckwheat; r. Spinach. Short dashed arrow indicates archaeology; long dashed arrow indicates evidence based on genetics data; solid arrow indicates genomics data; shaded area represents place of origin (Map from http://bzdt.ch.mnr.gov.cn/index.html)

Two hundred seven crops spanning 65 families, representing 41% of the estimated number of families in which domestication has occurred [19], were thought to have been distributed along the Land Silk Road (Table S1). Table 1 and Fig. 1 summarize the migration routes of 19 crops (16 genera, 13 families) for which genomic evidence is available along the Land Silk Road.

**Table 1:** The migration routes of 19 important crops according to archaeology, genetics, and genomics data

Species	Common Name	Ancient Book/Literature Record	Molecular Markers Evidence	Transcriptome/Genome Evidence
*Hordeum vulgare* var. *coeleste* L.	Tibetan barley	Tibet is the center of domestication.	Tibet is the center of domestication.	The Fertile Crescent→north Pakistan→India→Nepal→southern Tibet.
*Brassica juncea* (L.) Czern.	Mustard	China is the center of origin; Central Asia, Himalaya, and Middle East are the center of origin.		Mitochondrial genome evidence: vegetable mustard: China→India→Pakistan→Central Asia→the Middle East (along the Ancient Tea Horse Road and the Silk Road);
				SLAF-seq: China is the primary origin and diversity center.
				Genome-seq: a monophyletic origin in west Asia 8,000-14,000 years ago, and at least three subsequent independent domestication events in the last 500-5,000 years: seed mustard near Central Asia, oilseed mustard in the Indian subcontinent and root mustard in East Asia.
*Lactuca sativa* L.	Lettuce	Southwest Asia→Ancient Egypt→Ancient Greece and Rome→Europe→America.		RNA-seq: the Fertile Crescent→Europe (west); the Fertile Crescent→China (along the ancient Silk Road; east). Resequencing: the Caucasus→ancient Egypt→southern Europe→America.
*Fagopyrum esculentum* Moench	Buckwheat	Yunnan: to South East Asia, India, and Minor Asia in the 8^th^ century, to Siberia and Russia in the 13^th^ century, to Europe in the 15^th^ century, to the Americas in the 17^th^ century, and later to Africa. Ruins: 1) Xindian ruins, Fufeng county, Shanxi Province (Holocene); 2) Xishanping ruins, Tianshui City, Gansu Province (Holocene, 4,650-4,300 cal. BP); 3) Chenqimogou ruins, Lintan County, Gansu Province (Qijia culture, 4,000 cal. BP); 4) Yingpandi ruins near the Huangshui watershed (2,500 cal. BP); 5) Xueshan ruins, Chengjiang County, Yunnan Province (from the late Neolithic period to the Bronze Age, Shanzhai culture; 6) Haimenkou ruins, Jianchuan County, Yunnan Province (the bronze age； 7) Bayantala ruins, Chifeng City, Inner Mongolia Province (Liao dynasty, 916-1,123 AD); 8) Sunchangqing ruins, Baicheng City, Jilin Province (Liao and Jin dynasties, Liao dynasty 916-1,123 AD, Jin dynasty 1,115-1,234 AD); 9) Donghuishan ruins, Minle County, Gansu Province (3,610-3,458 years ago).	Southern China→northern China, Korean peninsula, and Japan; southern China→Bhutan, Nepal, Kashmir, as well as Karakuram and Hindukush [mainly through the southern slopes of the Himalaya region].	
*Cicer arietinum* L.	Chickpea	West/Central Asia→China. Near East, Central Asia, India, and Mediterranean were the first probable origin centers. South-eastern Turkey and adjoining Syria were the most probably origin place.		The Mediterranean/Fertile Crescent→Central Asia, and probably in parallel Central Asia→East Africa (Ethiopia) and South Aisa (India).
*Hordeum vulgare* L.	Barley	The Fertile Crescent→China. Western Asian Fertile Crescent→Central and Eastern Europe (west) along northern regions of the Mediterranean and Turkmenistan and Pakistan (east)→Eastern Central Asia and South Asia→Eastern Kazakhstan→Eastern China and Southern India.	The Fertile Crescent→China.	The Upper Jordan Valley→China.
*Triticum aestivum* L.	Wheat	The Fertile Crescent→China. Western Asian Fertile Crescent→Central and Eastern Europe [west] along northern regions of the Mediterranean and Turkmenistan and Pakistan [east] →Eastern Central Asia and South Asia→Eastern Kazakhstan→Eastern China and Southern India.		Iran→Central Asia→Altai Mountains→Qinghai, northern Tibet, middle and lower basin of the Yellow River, as well as Hexi Corridor (eastward).
*Ziziphus jujube* Mill.	Jujube	China→Korea→Japan/China→Europea→America		The Shanxi-Shaanxi area of China was primary domestication center and then disseminated into East China before finally extending into South China.
*Pistacia vera* L.	Pistachio	Central Asia (Native to the arid zones) → Iran (cultivated for 3000-4000 years) →Mediterranean Europe (by Romans).	Central Asia → Italy → Spain → other Mediterranean regions of Southern Europe→ North Africa→the Middle East→China→ the United States and Australia.	Originate from Central Asia and the Middle East.
*Brassica rapa* var. *rapa L.*	Turnip	Four possible origin place: Europeau-Central Asia, South Asia, East Asia, and Mediterranean Coast.	European–Central→Asia.	
*Medicago sativa* L.	Alfalfa	Media in ancient Persia (Central Asia, Caucasus, and Iran)→Greece→Europe, North Africa→the New World and Australia→all over the world.	Alfalfa originated from Southwest Asia, and was likely first domesticated in Caucasus, Turkey, and Iran over thousand years ago.	
*Juglans regia* L.	Walnut	West Asia→China.		
*Brassica oleracea* var. *botrytis L.*	Cauliflower	Mediterranean→China.		
*Vitis vinifera* L.	Grape	Near East→the South Caucasus→the western side of the Fertile Crescent, the Jordan Valley, and Egypt→Europe.	Near East is the origin place.	
*Spinacia oleracea* L.	Spinach	West Asia is the origin place.	*S. turkestanica* was the most likely ancestor of cultivated spinach, and spinach was introduced into China via Nepal after domestication.	Iran→North Africa and Europe→North America.
*Malus pumila* Mill.	Apple		Tianshan Mountains → Europe (along the Silk Road).	Tianshan Mountains → Europe (along the Silk Road).
*Cucumis sativus* L.	Cucumber	India is the Origin place.	India is the Origin place.	
*Morus alba* L.	Mulberry	Originate from China, including four major planting areae, Bayu region, Central Plains, Jiangnan Area, and the Pearl River Delta Region.		
*Pisum sativum* L.	Pea	Pea occurred Near East about 10, 000 years ago. Ethiopia, Mediterranean, Transcaucasia, western Asia, western Asia Minor were origin center, and Turkmen and Iran were secondary origin center.

### Crop species with conflicting evidence for their migration routes

There is conflicting evidence for the migration routes in each of five crops (Tibetan barley, mustard, lettuce, buckwheat, and chickpea). Tibetan barley is the main crop cultivated on the Tibetan Plateau for about 3,500 years [20]. Notably, there is highly controversial about its origin and migration, Tibet origin is the most concerned. Initially, six–rowed wild barley (*Hordeum agriocrithon* Åberg) was considered as wild species, which was found in Tibet and surrounding areas [21]. However, several studies noted that wild populations of six–rowed wild barley did not exist in Tibet, but were considered as weeds at the edges of fields [22–24]. Strikingly, cultivated barley originated from two–rowed wild barley, which was a true wild species with wild populations [25]. Around 7,000–10,000 years ago, humans began to grow domesticated two–rowed wild barley, which gradually produced six–rowed bottle–type wild barley and six–rowed sessile wild barley [25]. However, there are substantial differences between wild barley and Tibetan barley. The flowering and ripening stages are earlier in six–rowed wild barley than in Tibetan barley. In addition, the phenotypes of Tibetan wild barley and Tibetan barley are significantly different, for example, Tibetan wild barley sheds easily and has larger ear lobe [22]. Therefore, the present of wild barley in Tibet could not mean that Tibet was the center of origin or domestication. The uplift of Himalayan Mountains may mean that Central Asia was the sole route for wild barley migration between Near East and Tibetan Plateau [26]. Near East Fertile Crescent was identified as a primary center of origin of wild barley [26]. From this original center, wild barley spread to Central Asia and then migrated to the Tibetan Plateau, wild barley can adapt to harsh environments at high altitude [26]. In addition, agricultural development was one of the important driving factors for human movement to the Qinghai–Tibetan Plateau. There was evidence that Tibetan barley was introduced into China more than 5,200 years [27], when humans first inhabited the Qinghai–Tibetan Plateau [28]. Therefore, Near East is the most possible center of origin. 172 Whole–Genome Sequence (WGS) Tibetan barley accessions analysis also strongly supported that it was derived from eastern domesticated barley and subsequently introduced to Southern Tibet, most likely via North Pakistan, India, and Nepal [20].

Mustard is an important vegetable and oil crop. Four subspecies have been differentiated through long–term natural and artificial selection: *juncea* (seed mustard), *integrifolia* (leaf mustard), *napiformis* (root mustard), and *tumida* (stem mustard) [29]. However, mustard origin has been debated for decades. Chinese scholars insisted that China was the center of origin according to ancient records [30–34]. Meanwhile, mitochondrial genome evidence indicated that vegetable mustard originated from China and spread to India, Pakistan, Central Asia, and Middle East along the Ancient Tea Horse Road and the Silk Road [35]; 109 mustard accessions SLAF–seq (specific–locus amplified fragment sequencing) analysis also suggested that China was the primary center of origin and diversity [36]. However, some scholars proposed that Central Asia, Himalaya, and Middle East were the centers of origin [37–43]. Furthermore, whether *B. juncea* has a monophyletic or polyphyletic origin is also uncertain. Morphological evidence and 109 SLAF–seq accessions analysis suggested a single origin [30, 31, 36], while more evidence including chemotaxonomy [38], nuclear DNA markers [44, 45], and chloroplast genomic markers [46] suggested a polyphyletic origin. Population genomics provides an opportunity to understand crops origin and domestication [47]. 480 accessions genome re–sequencing as well as archaeological evidence indicated that mustard was monophyletic origin in West Asia 8,000–14,000 years ago, and three subsequent independent domestication event at last 500–5,000 years: seed mustard near Central Asia, oilseed mustard in the Indian subcontinent, and root mustard in East Asia [48]. These results conflicted with records from ancient culture sites, unearthed relics, and historical documents. It was difficult to accurately identify vegetables and fruits at ancient culture sites and unearthed relics. Images at ancient culture sites and on unearthed relics could be abstract. Additionally, the reliability of images depended on the quality and quantity. If only one painter depicted one image in single painting, this image may be unreliable [49].

Lettuce is one of the most important vegetables worldwide. It has a long history of cultivation, including leafy lettuce, stem lettuce, and oil lettuce. *Lactuca serriola* L. was considered as the wild progenitor [50, 51]. RNA sequencing analyses of 240 wild and cultivated accessions showed that lettuce underwent a single domesticated event from wild *L. serriola* L., and that cultivated lettuce originated in the Fertile Crescent more than 10,800 years, consistent with the historical records of the beginning of human–associated plant domestication about 12,000 years ago [52]. Recently, whole–genome resequencing of 445 *Lactuca* accessions revealed the domestication history of cultivated lettuce. The results clarified that the Caucasus was probably the domestication center of lettuce around 4,000 BC, and later lettuce spread to Ancient Egypt, and Southern Europe in ancient Roman [53]. Surprisingly, other studies reached different conclusions about its origin and distribution. According to the first record on the walls of Egyptian tombs at around 2,500 BC, lettuce spread from Southwest Asia to Ancient Egypt more than 4,500 years [54]. It was successively introduced from Ancient Egypt into Ancient Greece and Rome, Europe, and America based on morphological characters and ancient book records [50, 54, 55]. A study in 1990 suggested that lettuce also originated from Southwest Asia, in the region between Egypt and Iran, with the highest number of related wild species [56]. However, the first wild species of lettuce has been identified in 1997 and 2008, therefore, Southwest Asia Origin may be incorrect.

Buckwheat plays an important role in the dietary structure due to its rich in fatty acids, essential amino acids, and vitamins [57]. Early archaeological records suggested that wild buckwheat grew in Yunnan; cultivated buckwheat spread from its original place to other parts of the world: to South East Asia, India, and Minor Asia in the eighth century, to Siberia and Russia in the thirteenth century, to Europe in the fifteenth century, to the Americas in the seventeenth century, and later to Africa [58]. There is another way to say that buckwheat may originate from Southern China and moved westward along the southern slopes of Himalayas [59–61]. However, Wang and Lu indicated that wild species was widely distributed in the Qinghai–Tibetan Plateau, Loess Plateau, Western Sichuan Plateau, Yunnan–Guizhou Plateau, as well as Western Hunan and Hubei [62]. Furthermore, RAPD markers analyses of 29 buckwheat landraces in Asia revealed other diffusion routes: one major route was from Southern China, to Northern China, Korean peninsula, and Japan; and the other to the Himalayan region, mainly through the southern slopes of the Himalayas, with the exact route from Southern China, to Bhutan, Nepal, Kashmir, and Karakoram and Hindu Kush [63, 64]. During the last 10 decades of archeological research, the remains of buckwheat seeds, especially prehistoric archeological discoveries, have rarely been found. Surprisingly, buckwheat pollen was respectively found at Xishanping ruins (4,650–4,300 cal. BP; Tianshui City, Gansu Province) in Holocene [65] and Xindian ruins (Fufeng county, Shanxi Province) in Holocene [66]. In 2010, buckwheat starch was separated from human dental calculus in the Chenqimogou ruins (Qijia culture, 4,000 cal. BP; Lintan County, Gansu Province) [67]. Excitingly, buckwheat first appear in the Yingpandi ruins (2,500 cal. BP) near the Huangshui watershed, as one buckwheat kernel was found here [68]. Later, buckwheat kernels were found in the Xueshan ruins (Chengjiang County, Yunnan Province; from the late Neolithic period to the Bronze Age, Shanzhai culture), in the Haimenkou ruins (Jianchuan County, Yunnan Province; the bronze age) [69], in the Bayantala ruins (Chifeng City, Inner Mongolia Province; Liao dynasty, 916–1,123 AD) [70], and in the Sunchangqing ruins (Baicheng City, Jilin Province; Liao and Jin dynasties, Liao dynasty 916–1,123 AD, Jin dynasty 1,115–1,234 AD) [71], respectively. Notably, three buckwheat kernels were found in Donghuishan ruins (Minle County, Gansu Province). The ^14^C dating result showed that it was from 3,610–3,458 years ago, these carbonized buckwheat kernels from the late Neolithic period were the oldest that had been found in China; these findings provided new evidence that buckwheat originated from the Qinghai–Tibetan Plateau [72]. These need to be further verified by re–sequencing on the basis of the buckwheat genome [73].

Chickpea (*C. arietinum* L.) is the second widely grown legume crop after soybean, mainly growing in South Asia. Archaeological records suggested that the probably original centers of chickpea were Near East, Central Asia, India, Mediterranean, and Southwest Africa (Fertile Crescent) [74, 75]. However, some researchers have proposed South–eastern Turkey and adjoining Syria as likely places of origin [76, 77]. Consistent with this, wild chickpea was found in 10 locations within a small area in Southeastern Turkey and Northern Syria [78]. 28 chickpea accessions AFLP markers indicated that three main centers of diversity were Pakistan–Afghanistan, Iran–Turkey, and Syria–Lebanon [79]. Furthermore, resequencing of 429 chickpea accessions revealed that the Eastern Mediterranean was the primary center of origin and the migration route was from the Mediterranean/Fertile Crescent to Central Asia, and then probably in parallel from Central Asia to East Africa (Ethiopia) and South Asia (India) [80]. This was inconsistent with above archaeological evidence. Morphological characters in wild species have many limitations, e.g., low polymorphism, low heritability, and late expression [81].

Crop exchange is very active and important for early humans, there are different records for the one crop species in different period. At least five conflict events have been suggested along the Land Silk Road (Fig. 1). Previous studies about domestication mainly concentrated on morphological, archeological, and agronomic aspects, utilization of molecular markers have also provided evidence for crop migration. However, incomplete early knowledge may have obscured the details of the domestication process, for example, ambiguous wild species, unreliable ancient culture sites, unearthed relics records, complex phenotypic variations, and limited sample sizes. In the present study, Tibetan barley, mustard, lettuce, and chickpea have been confirmed by genomic studies, which have provided the most convincing evidence by comprehensive germplasm collection and high genetic diversity. Genomic re–sequencing study can better understand population structure of germplasm, domestication, and post–domestication divergence.

### Crop species with consistent evidence for their migration routes

Early crop globalization is one of the most magnificent events in human social development. The most important and widely influential event is the exchange of east millet agriculture and west Asia wheat/barley agricultural system. Barley and wheat are the founding crops of agriculture in the ancient Near East and Europe [82]. Domesticated barley and wheat were present in archaeological records at least 10,000 years ago [82]. Morphological and population genetic analyses verified that barley and wheat were domesticated in the Fertile Crescent, where their wild relatives still thrive today [83–85]. On the basis of book record and the available radiocarbon dates, lots of barley and wheat spread from Western Asian Fertile Crescent westwards across Central and Eastern Europe and along northern regions of Mediterranean [82]; to the east, various types were recorded in Turkmenistan and Pakistan before 5,000 BC [86]. Furthermore, barley and wheat cultivation moved into Eastern Central Asia and South Asia at 5,000–2,500 BC [87], these crops occurred in Eastern Kazakhstan by 2,500 BC [88], the Indus region and in the upper Ganges [89]. Later, the Fertile Crescent barley and wheat expanded into Eastern China and Southern India at 2,500–1,500 BC [87]. Genome analysis of five 6,000–year–old barley samples clarified that domesticated barley originated from the Upper Jordan Valley, in fact, the 6,000–year–old domesticated barley was remarkably similar to proximate extant landraces, indicating that the major domestication event had already occurred by that time [90]. Archaeobotanical, palynological, and anthracological data revealed that wheat arrived at west Tianshan Mountains in Central Asia around 5,500 years ago, and then spread into the Altai region about 5,200 years ago [27]. It is a remarkable fact that barley and wheat are introduced into China before the Silk Road.

Jujube and pistachio, endemic food in Xinjiang (China), are important perennial tree with economic, nutritional, and medicinal value. Both crops have been genomic re–sequencing analysis, the domestication routes are consistent with previous archaeological records and molecular evidence. Jujube has been cultivated 7,000 years; it was introduced from China to Korea, Japan and other neighbouring countries around 100 BC and then dispersed to Europe along the Silk Road according to archaeological and book records [91, 92]. Population genomic analyses clarified that Shanxi–shaanxi area of China was primary domestication center for jujube, and that it then spread to East China before finally extending into South China. Genomic analyses also revealed that *Ziziphus acidojujjuba* and *Z. jujuba* diverged around 2.7 Mya, indicating that there was a long pre–domestication period prior to human intervention [93]. This suggested that jujube spread was earlier than the Silk Road. Pistachio originated from the arid zones of Central Asia, cultivated for 3,000–4,000 years in Iran, and then spread into Mediterranean by Romans at the early Christian era based on ancient records [94]. Genetic analyses suggested that pistachio cultivation migrated westward from Central Asia to Italy, Spain, and other Mediterranean and southern European regions, to north Africa, the Middle East, and China, and then to the United States and Australia [95, 96]. Whole genome and transcriptome analyses supported the results of ancient records and genetic analysis that pistachio originated in Central Asia and the Middle East, and that, wild and domestic species diverged about 8,000 years ago [97]. Consistent with this, archeological records showed that pistachio seeds were a common food as early as 6,750 BC [97]. However, the relationship between the time of the spread to China and the Silk Road was still unclear.

For another ten crops, spinach and apple have been genomic re–sequencing analysis; alfalfa, turnip, walnut, cauliflower, grape, cucumber, mulberry, and pea are only one or few lines of evidence for the domestication route. The relationship between turnip domestication route and the Silk Road is still ambiguous, whereas alfalfa, walnut, spinach, grape, pea, apple, cauliflower, mulberry, and cucumber are introduced into China with trade and human migration along the Silk Road [12]. Four possible origin places of turnip were Europe–Central Asia, South Asia, East Asia, and Mediterranean coast [98–101]. Furthermore, transcriptome analyses indicated that it originated in Europe–Central Asia, and was then introduced into Asia around 2,400–4,100 years ago [102]. Alfalfa originated from Media in ancient Persia (i.e., Central Asia, Caucasus, and Iran) [103], introduced into Greece about 490 BC, and later acquired by the Romans based on archaeological evidence [104]. As the military operations of the Roman Empire proceeded, alfalfa was the best fodder to feed warhorses and brought to many regions of Europe, North Africa, and further eastward [104]. In the sixteenth and eighteenth centuries, European colonists carried alfalfa to the New World and Australia, so that this forage crop is now distributed almost all around the world [105, 106]. Nuclear polymorphisms analyses indicated that alfalfa originated in Southwest Asia, and first domesticated in Caucasus, Turkey, and Iran over thousand years ago [107]. Evidently, walnut and spinach originated from West Asia. Walnut origin was based on historical record [75], it traded along the Silk Road and overcame geographical barriers to move across Asia [108]. Spinach was from Pyrenees mountain at the late 12^th^ or early thirteenth century, or native to Central Asia and originated in Iran based on archaeological records [109, 110]. Phylogenetic and population structure analyses indicated that *S. turkestanica* was the most likely ancestor of cultivated spinach, and spinach was introduced into China via Nepal after domestication; however, it remains obscure how spinach was introduced into Nepal [111]. Transcriptome sequencing of 120 cultivated and wild spinach accessions confirmed that spinach was native to Iran, and was introduced to North Africa and Europe before being brought to North America [112]. Remarkably, grape and pea originated from Near East. Cultivated grape was domesticated from the wild progenitor *V. vinifera* subsp. *sylvestris* in the Near East at 6,000–8,000 years ago based on archaeological records. After domestication, cultivated grape was present in South Caucasus between Caspian and Black Seas, and then spread south to the western side of Fertile Crescent, Jordan Valley, and Egypt around 5,000 years ago, finally reached Western Europe about 2,800 years ago [113, 114]. Genetic evidence also supported the origin of grape in the Near East [115]. Pea occurred in Near East about 10,000 years ago [116, 117]. Its primary origin centers were Ethiopia, Mediterranean, Transcaucasia, Western Asia, and Western Asia Minor, while its secondary origin center were Turkmenistan and Iran [118]. Apple domestication was driven by different wild species hybridization, it may migrate from Tianshan Mountain to Europe along the Silk Road based on genetic and genomic evidence [119, 120]. Cauliflower spread from Mediterranean to China according to ancient book [121]. Genomic evidence verified that cultivated cauliflower diverged from the ancestral *B. oleracea* about 3 Mya, but it did not provided any information about the migration route [122]. Mulberry originated in China, and cultivated in the Bayu region, Central Plains, Jiangnan area, and Pearl River Delta region [123, 124]. However, 134 resequencing accessions analysis classified domesticated mulberry into three geographical groups, that is, the Taihu Basin of Southeastern China (Hu mulberry), Northern and Southwestern China, and Japan [125].Wild cucumber was present in India and domesticated in Asia about 3,000 years ago using nuclear and plastid markers [126], consistent with the results of DNA analyses in archaeological specimens [127].

A synthesis of plant archaeology, genetics, and genomics can generate new perspectives about how domestication proceeds [47, 128]. In the last few years, combing with archaeological and genetic research has led to a greater understanding of the mode and tempo of domestication [129]. Here we discuss 15 crops that is consistent evidence for the migration routes, among them, each of 8 crops have been proved based on genetic and archaeological evidence. It is clear that genomics have solidified genetic and archaeological evidence. These species are relatively young; domestication occurs in the Pleistocene–Holocene during which are global warming period after the last glacial, first in the Fertile Crescent and in other early centers of agriculture [129]. For example, west Asia was home to barley, wheat, walnut, grape, spinach, and pea—crops that are still among the most valuable crops for food and feed in the modern world. Humans in West Asia domesticated these species and became the world’s first farmer around 8,500 BC [130]. From around that time, the switch from the hunting lifestyle to food processing allowed humans to establish permanent settlements instead of migrating to explore wild food supplies [131]. By 4,000 years ago, ancient humans have domesticated major crops upon which human survival is still dependent, including barley and wheat.

## Conclusion

Early crop globalization is an important event in human social development. The Land Silk Road is the main pathway for the exchange of eastern and western cultures and civilizations. In the present study, we have synthesized archaeology, genetics, and genomics to trace the migration process of crops along the Land Silk Road. The migration routes of 19 crops for which genome evidence is available. There is conflicting evidence for the migration in each of five crops (Tibetan barley, mustard, lettuce, buckwheat, chickpea), relatively consistent evidence for the migration in each of 14 crops (barley, wheat, pistachio, jujube, alfalfa, turnip, walnut, cauliflower, grape, spinach, apple, cucumber, mulberry, pea). Remarkably, incomplete early knowledge (ambiguous wild species, unreliable ancient culture sites, unearthed relics records, complex phenotypic variations, and limited sample sizes), record differences in different period, and data analysis techniques effect the understanding of the migration process. Notably, it is clear that genomics can solidify genetic and archaeological evidence.

The relationships between the 19 crops migration process and the Land Silk Road have also been clarified. Tibetan barley, barley, wheat, and jujube were introduced into China before the Silk Road; while mustard, lettuce, buckwheat, chickpea, alfalfa, walnut, cauliflower, grape, spinach, apple, cucumber, mulberry, and pea were introduced into China with trade and human migration along the Silk Road. However, relationships between the Silk Road and the spread of turnip and pistachio is still ambiguous. We note that a limited number of crops are discussed in this study. More research, especially on distribution of crops along the Maritime Silk Road, is needed to make more robust conclusions.

## Supplementary Information

**Additional file 1.**

**Additional file 2:** **Table S1.** Two hundred seven domesticated plant species spanning 65 taxonomic families that were spread along the Land Silk Road.

## Data Availability

All data generated or analysed during this study are included in this published article [and its supplementary information files].
