# Correction: Exchanges of economic plants along the land silk road

**DOI:** 10.1186/s12870-023-04080-7

**Published:** 2023-02-02

**Authors:** Guangyan Wang, Qian Chen, Ya Yang, Yuanwen Duan, Yongping Yang

**Affiliations:** 1 Germplasm Bank of Wild Species, Kunming Institute of Botany, Chinese Academy of Sciences, Kunming, 650201 China; 2 Institute of Tibetan Plateau Research at Kunming, Chinese Academy of Sciences, Kunming, 650201 China

**Correction: BMC Plant Biol 22, 619 (2022)**

**https://doi.org/10.1186/s12870-022-04022-9**

Following the publication of the original article [1], author would like to correct some errors in the Conclusion part of the Abstract section and in the paragraph 2 part of the Conclusion section.

The incorrect lines in the Conclusion part of the Abstract section and in the paragraph 2 part of the Conclusion section are:

1. There was strong evidence that Tibetan barley, barley, wheat, and jujube were introduced into China before the existence of the Land Silk Road; and mustard, lettuce, buckwheat, chickpea, alfalfa, walnut, cauliflower, grape, spinach, apple, cucumber, mulberry, and pea spread to China via trade and human migration along the Land Silk Road.
2. Tibetan barley, barley, wheat, and jujube were introduced into China before the Silk Road; while mustard, lettuce, buckwheat, chickpea, alfalfa, walnut, cauliflower, grape, spinach, apple, cucumber, mulberry, and pea were introduced into China with trade and human migration along the Silk Road.

The correct lines are:

1. There was strong evidence that Tibetan barley, barley, wheat, and jujube were spread before the existence of the Land Silk Road; and mustard, lettuce, buckwheat, chickpea, alfalfa, walnut, cauliflower, grape, spinach, apple, cucumber, mulberry, and pea were spread via trade and human migration along the Land Silk Road.
2. Tibetan barley, barley, wheat, and jujube were spread before the Silk Road; while mustard, lettuce, buckwheat, chickpea, alfalfa, walnut, cauliflower, grape, spinach, apple, cucumber, mulberry, and pea were spread with trade and human migration along the Silk Road.

Moreover, another error was spotted in the body of the article. The line ‘whereas alfalfa, walnut, spinach, grape, pea, apple, cauliflower, mulberry, and cucumber are introduced into China with trade and human migration along the Silk Road.’ should be corrected to ‘whereas alfalfa, walnut, spinach, grape, pea, apple, cauliflower, mulberry, and cucumber are spread with trade and human migration along the Silk Road.’

The original article [1] has been corrected.
