# Supplementary material for: Exchanges of economic plants along the land silk road
Source: BMC Plant Biol. 2022 Dec 29;22:619. doi: 10.1186/s12870-022-04022-9 (PMC9801618; doi:10.1186/s12870-022-04022-9)
Supplement: Supplementary file 1 — Additional file 1. [file 12870_2022_4022_MOESM1_ESM.docx]

**Data acquisition**

The crop species discussed in this paper were selected from those described in literature reviews on economic plants in “One Belt and One Road” (Chapter Central Asia, West Asia, Mediterranean) [[1](#_ENREF_1), [2](#_ENREF_2)]. Books/literature records, molecular markers, and genomic studies explore the migration pattern of 19 crops (16 genera, 13 families) along the Lank Silk Road.

We focused on 19 important crops. 1) Tibetan barley (*Hordeum vulgare* L., qingke) is one of the founder crops of Old World agriculture and probably the first crop cultivated by humans [[3](#_ENREF_3)]. Importantly, it is a major food of Tibetans [[4](#_ENREF_4)]. 2) Mustard (*Brassica juncea* (L.) Czern.) is an important part of the U’s triangle model of *Brassica* and comprises vegetable–types and oil–types [[5](#_ENREF_5)]. The different types of mustard differentiated after a long period of natural selection and artificial selection, including *B. juncea* var. *tumida* Tsen et Lee, *B. juncea* var. *megarrhiza* Tsen et Lee, and *B. juncea* var. *multicep* Tsen et Lee. 3) Lettuce (*Lactuca sativa* L.) is an important vegetable worldwide and has six types: butterhead, crisphead, looseleaf, romaine, stem, and oilseed. 4) Buckwheat (*Fagopyrum esculentum* Moench) is a widely cultivated annual crop. Its grain contains high levels of starch, protein, flavonoids, and dietary fiber [[6](#_ENREF_6)]. Buckwheat flour is gluten–free and can replace wheat flour in a coeliac diet [[7](#_ENREF_7)]. 5) Chickpea (*Cicer arietinum* L.) is the world’s second most widely grown legume, and its seeds are a primary source of protein in the human diet [[8](#_ENREF_8)]. 6) Barley (*Hordeum vulgare* L.) is one of the most important early cereals, and the founder agricultural crop in the ancient Near East and Europe [[9](#_ENREF_9)]. It has been used food and feed, for brewing, and in medicine. 7) Wheat (*Triticum aestivum* L.) is the most widely cultivated crop worldwide. It contributes about a fifth of the total calories consumed by humans and provides more protein than any other food [[10](#_ENREF_10)]. Importantly, wheat yields affect the global economy [[11](#_ENREF_11)]. 8) Pistachio (*Pistacia vera* L.), a desert plant that is highly tolerant of saline soil, is one of the most important commercial nut crops. Pistachio is the fifth largest nut crop worldwide, and Iran and the USA are the major producers [[12](#_ENREF_12)]. 9) Jujube (*Ziziphus jujube* Mill.) is a popular fruit tree with important economic, ecological, and nutritional value. It is also used as a traditional herbal medicine [[13](#_ENREF_13)]. 10) Alfalfa (*Medicago sativa* L.), the Queen of Forage, is a perennial legume that provides inexpensive, nutritious, and highly digestible forage. It is grown worldwide [[14](#_ENREF_14)]. 11) Turnip (*Brassica rapa* var. *rapa* L.) is one of the most important vegetables worldwide, and is used as food and fodder, and in medicines [[15](#_ENREF_15)]. 12) Walnut (*Juglans regia* L.) grows in temperate regions around the world. It is an economically important tree species that is grown for its timber and edible nuts [[16](#_ENREF_16)]. 13) Cauliflower (*Brassica oleracea* L. var. *botrytis* L.) is an important variety of *B. oleracea*. It differs from most *Brassica* species in that it forms a specialized organ called the curd [[17](#_ENREF_17)], which consists of indeterminate inflorescences and shortened inflorescence branches [[18](#_ENREF_18), [19](#_ENREF_19)]. It is the primary edible organ of cauliflower, and contains abundant fiber and nutrients, including sulforaphane [[20](#_ENREF_20)]. 14) Grape (*Vitis vinifera* L.) is the most economically important horticultural crop in the world. It is one of the earliest domesticated fruit crops, and is widely cultivated for the fruit and the wine produced from it [[21](#_ENREF_21)]. 15) Spinach (*Spinacia oleracea* L.), an important and nutritious green leafy vegetable, is a rich source of carotenoids, folic acid, vitamin C, calcium, and iron. It can be used as a salad leaf, a cooked vegetable, or as an ingredient in other dishes [[22](#_ENREF_22)]. 16) Apple (*Malus domestica* Borkh.) is a popular fruit in temperate regions of the world. 17) Cucumber (*Cucumis sativus* L.) is an economically important vegetable crop worldwide, and one of the top five vegetable crops worldwide. Cucumber is consumed fresh or processed into pickles [[23](#_ENREF_23), [24](#_ENREF_24)]. 18) Mulberry *(Morus alba* L.), a deciduous tree, is grown as the sole food source for silkworm (*Bombyx mori*), and is therefore essential for the silk industry [[25](#_ENREF_25), [26](#_ENREF_26)]. 19) Pea (*Pisum sativum* L.) is an important legume crop that is not only grown for food and feed, but also has significant ecological advantages in the sustainable development of agriculture [[27](#_ENREF_27)].

**Migration routes**

For each of five crops (Tibetan barley, mustard, lettuce, buckwheat, and chickpea), there is conflicting evidence for their migration routes based on ancient books/literature, molecular marker data, and genomic evidence (Fig. 1). 1) Tibetan barley: Tibet was potentially the center of domestication according to ancient records, phylogenetic tree based on 1309 DArT markers with 238 accessions, and population–based phylogenetic analysis using 103 accessions [[28–31](#_ENREF_28)]. However, genomic evidence suggested that qingke derived from eastern domesticated barley and was introduced to Southern Tibet, most likely *via* north Pakistan, India, and Nepal [[32](#_ENREF_32)]. 2) Mustard: some Chinese scholars suggested that China was the center of origin based on ancient book records [[33–37](#_ENREF_33)]; and other researchers have proposed that mustard originated from Central Asia to the Himalayas [[38](#_ENREF_38), [39](#_ENREF_39)]. Mitochondrial genome evidence indicated that vegetable mustard originated in China and spread to India, Pakistan, Central Asia, and the Middle East along the Ancient Tea Horse Road and the Silk Road [[40](#_ENREF_40)]. Another study used a specific–locus amplified fragment sequencing (SLAF–seq) strategy to analyse 109 mustard accessions from China, India, Australia, and European countries. Their results suggested that China was the primary origin and center of diversity, and that mustard was a monophyletic origin and evolved into vegetable and oilseed varieties [[5](#_ENREF_5)]. Genome re–sequencing evidence showed that mustard was monophyletic origin in West Asia 8,000–14,000 years ago, and three subsequent independent domestication event in the at last 500–5,000 years: seed mustard near Central Asia, oilseed mustard in the Indian subcontinent, and root mustard in East Asia [[41](#_ENREF_41)]. 3) Lettuce: Based on the first record on the walls of Egyptian tombs, lettuce spread from Southwest Asia to ancient Egypt more than 4,500 years [[42](#_ENREF_42)]. Records in ancient books and analyses of its morphological characters suggested that it was then introduced into ancient Greece and Rome, Europe, and America from Ancient Egypt [[42–44](#_ENREF_42)]. The results of RNA sequencing analyses verified that lettuce underwent a single domestication event from wild *L. serriola* L., and that cultivated lettuce originated in the Fertile Crescent more than 10,800 years [[45](#_ENREF_45)]. Whole–genome resequencing of 445 accessions revealed that the Caucasus was probably the center of domestication of lettuce. After being domesticated by humans, it spread into ancient Egypt, and then into Southern Europe in Roman times [[46](#_ENREF_46)]. 4) Buckwheat: Early archaeological and historical records indicated that wild buckwheat was present in Yunnan; and that cultivated buckwheat spread from its place of origin to South East Asia, India, and Minor Asia in the 8^th^ century; to Siberia and Russia in the 13^th^ century; to Europe in the 15^th^ century; to the Americas in the 17^th^ century; and later to Africa [[47](#_ENREF_47)]. However, analyses based on RAPD markers revealed different dispersal routes of buckwheat: one major route was from Southern China, to Northern China, Korean peninsula, and Japan; the other one was the Himalaya region, mainly through the southern slopes of the Himalaya region, with the exact route from Southern China, to Bhutan Nepal, Kashmir, and Karakoram and Hindu Kush [[48](#_ENREF_48)]. During the last 10 decades of archeological research, buckwheat pollen was found at Xindian ruins, Fufeng county, Shanxi Province in Holocene [[49](#_ENREF_49)]. It was also found at Xishanping ruins (4,650–4,300 cal. BP), Tianshui City, Gansu Province in Holocene [[50](#_ENREF_50)]. Buckwheat starch was separated from human dental calculus in the Chenqimogou ruins (Qijia culture, 4,000 cal. BP), Lintan County, Gansu Province [[51](#_ENREF_51)]. Buckwheat may first appear in the Yingpandi ruins (2,500 cal. BP) near the Huangshui watershed, as one buckwheat kernel was found here [[52](#_ENREF_52)]. Buckwheat kernels were found in the Xueshan ruins (from the late Neolithic period to the Bronze Age, Shanzhai culture), Chengjiang County, Yunnan Province, in the Haimenkou ruins (the bronze age), Jianchuan County, Province [[53](#_ENREF_53)], in the Bayantala ruins (Liao dynasty, 916–1,123 AD), Chifeng City, Inner Mongolia Province [[54](#_ENREF_54)], and in the Sunchangqing ruins (Liao and Jin dynasties, Liao dynasty 916–1,123 AD, Jin dynasty 1,115–1,234 AD), Baicheng City, Jilin Province [[55](#_ENREF_55)], separately. Three buckwheat kernels were found in Donghuishan ruins, Minle County, Gansu Province. The ^14^C dating result showed that it was from 3,610–3,458 years before present [[56](#_ENREF_56)]. 5) Chickpea: Ancient book records indicated that chickpea spread from West/Central Asia to China [[1](#_ENREF_1)]. Analyses based on whole–genome resequencing of 429 lines sampled from 45 countries suggested that the Eastern Mediterranean was the primary center of origin and that the migration route was from the Mediterranean/Fertile Crescent to Central Asia, and probably in parallel to Central Asia from East Africa (Ethiopia) and South Asia (India) [[57](#_ENREF_57)].

For each of another 14 crops (barley, wheat, jujube, pistachio, turnip, alfalfa, walnut, cauliflower, grape, spinach, apple, cucumber, mulberry, pea) have consistent evidence for their migration routes based on archaeology, genetics, and genomic evidence. 1) Book record and the available radiocarbon dates showed that a number of barley and wheat spread from Western Asian Fertile Crescent westwards across Central and Eastern Europe and along northern regions of the Mediterranean [[58](#_ENREF_58)]; to the east, various types were recorded in Turkmenistan and Pakistan before 5,000 BC [[59](#_ENREF_59)]. Barley and wheat cultivation moved into Eastern Central Asia and South Asia at 5,000–2,500 BC [[60](#_ENREF_60)], these crops occurred in Eastern Kazakhstan by 2500 BC [[61](#_ENREF_61)], the Indus region and in the upper Ganges [[62](#_ENREF_62)]. Later, the Fertile Crescent barley and wheat expanded into Eastern China and Southern India at 2,500–1,500 BC [[60](#_ENREF_60)]. The results of morphological, population genetic, and genomic analyses suggested that barley and wheat originated in the Fertile Crescent and then spread to China [[3](#_ENREF_3), [58](#_ENREF_58), [63–65](#_ENREF_63)]. 2) Ancient records indicated that jujube was native to China, and introduced into Korea, Japan and other neighbouring countries around 100 BC and then dispersed to Europe and America along the Silk Road [[66](#_ENREF_66), [67](#_ENREF_67)]. Genomic analyses showed that Shanxi–Shaanxi area of China was its primary domestication center and then it spread to East China before finally extending into South China [[13](#_ENREF_13)]. 3) Pistachio originated from the arid zones of Central Asia. It had been cultivated for 3,000–4,000 years in Iran, and was introduced into the Mediterranean region by Romans in the early Christian era [[68](#_ENREF_68)]. Genetic analyses suggested that pistachio cultivation migrated westward from Central Asia to Italy, Spain, and other Southern Mediterranean regions of Southern Europe, to north Africa, the Middle East, and China, and then to the United States and Australia [[69](#_ENREF_69), [70](#_ENREF_70)]. Whole genome and transcriptome analyses revealed that pistachio originated from Central Asia and the Middle East. The wild and domestic pistachio species diverged about 8,000 years ago, consistent with archaeological records showing that pistachio seeds were a common food as early as 6,750 BC. Pistachio included a two–step domestication processes, with initial domestication followed by improvement through crop breeding. 4) According to ancient evidence, alfalfa originated from Media in ancient Persia, i.e., Central Asia, Caucasus, and Iran [[71](#_ENREF_71)]. Its two probable centers of origin were Central Asia and the region covering Caucasus, Asia Minor, and Northwest Iran [[72](#_ENREF_72)]. In about 490 BC, alfalfa was introduced into Greece by invading Persians and was later acquired by the Romans. As the military operations of the Roman Empire proceeded, alfalfa was the best fodder to feed warhorses and so it spread to many regions of Europe, North Africa, and further eastward. In the 16^th^ and 18^th^ centuries, European colonists took alfalfa to the New World and Australia. Consequently, this forage crop has spread around almost the entire world [[73](#_ENREF_73), [74](#_ENREF_74)]. The genomic evidence was consistent with the migration and cultivation history of alfalfa, and indicated that it was brought to South America in the 16^th^ century and was later cultivated in North America [[75](#_ENREF_75)]. 5) Turnip: The possible centers of origin were Europe–Central Asia, South Asia, East Asia, and the Mediterranean coast [[76–79](#_ENREF_76)]. Transcriptome analysis indicated that turnip originated from Europe–Central Asia, and was introduced into Asia around 2,400–4,100 years ago [[80](#_ENREF_80)]. 6) Walnut: Historical records showed that walnut was introduced from West Asia into China [[1](#_ENREF_1)]. Analyses of morphological and molecular variations indicated that the place of origin of walnut was probably South Asia [[81](#_ENREF_81)]. 7) Cauliflower: Ancient book records indicated that cauliflower originated from the Mediterranean and spread to China [[82](#_ENREF_82)]. 8) Grape: According to archaeological records, cultivated grape originated in the Near East, and then spread southwards to the western side of the Fertile Crescent, the Jordan Valley, and Egypt around 5,000 years ago, and finally reached Western Europe around 2,800 years ago [[83](#_ENREF_83), [84](#_ENREF_84)]. 9) Spinach: The archaeological record indicated that spinach was native to Central Asia and originated from Iran [[22](#_ENREF_22)]. Analyses based on genetic markers suggested that *Spinach turkestanica* was the most likely ancestor of cultivated spinach, and that spinach was introduced into China *via* Nepal after domestication [[85](#_ENREF_85)]. Transcriptome sequencing of 120 cultivated and wild spinach accessions revealed that it was native to Iran, and was introduced to North Africa and Europe, and then brought to North America [[86](#_ENREF_86)]. 10) Apple: The results of genetic and genomic analyses indicated that apple spread from the Tianshan Mountains to Europe along the Silk Road [[87](#_ENREF_87), [88](#_ENREF_88)]. 11) Cucumber: Analyses of DNA extracted from archaeological specimens as well as nuclear and plastid markers showed that wild cucumber grew in India and was domesticated in Asia about 3,000 years ago [[89](#_ENREF_89), [90](#_ENREF_90)]. 12) Mulberry: According to ancient records, mulberry originated from China, and there were four major growing areas: the Bayu region, Central Plains, the Jiangnan area, and the Pearl River Delta region [[25](#_ENREF_25), [26](#_ENREF_26)]. Population genomic analyses based on resequencing of 134 mulberry accessions showed that *M. alba* was a diploid with 28 chromosomes, and that multiple domesticated mulberry accessions previously classified as different species actually belonged to one species [[91](#_ENREF_91)]. 13) Pea: Ancient documents record pea in the Near East about 10,000 years ago [[92](#_ENREF_92), [93](#_ENREF_93)]. Its centers of origin were Ethiopia, Mediterranean, Transcaucasia, Western Asia, and Western Asia Minor, and its secondary centers of origin were Turkmenistan and Iran [[94](#_ENREF_94)]. However, genomic evidence has not revealed the domestication process of pea [[95](#_ENREF_95)].

**References**

1. Yuan CQ, Shu CJ, Wang M, Xiao ZC, Zhang WM. Application and development of economic plants in "One Belt and One Road" (Chapter Central Asia, West Asia). Chinese Wild Plant Res. 2016;35(2):3–13.

2. Yuan CQ, Shu CJ, Lu LQ, Xiao ZC, Zhang WM. Application and development of economic plants in "One Belt and One Road" (Chapter Europe, Mediterranean Region). Chinese Wild Plant Res. 2016; 35(2):5–6.

3. Badr A, Müller K, Schäfer–Pregl R, Rabey HE, Effgen S, Ibrahim HH, et al. On the origin and domestication history of barley (*Hordeum vulgare*). Mol Biol Evol. 2000;17(4):499–510.

4. Ma DQ, Xu TW. The research on classification and origin of cultivated barley in Tibet Autonomous Region. Scientia Agri Sinica. 1988;21(5):7–14.

5. Yang J, Zhang C, Zhao N, Zhang L, Hu Z, Chen S, et al. Chinese root–type mustard provides phylogenomic insights into the evolution of the multi–use diversified allopolyploid *Brassica juncea*. Mol Plant. 2018;11(3):512–4.

6. Gimenezbastida JA, Zielinski H. Buckwheat as a functional food and its effects on health. J Agr Food Chem. 2015;63(36):7896–913.

7. Comino I, Moreno Mde L, Real A, Rodriguez–Herrera A, Barro F, Sousa C. The gluten–free diet: testing alternative cereals tolerated by celiac patients. Nutrients. 2013;5(10):4250–68.

8. Jukanti AK, Gaur PM, Gowda CL, Chibbar RN. Nutritional quality and health benefits of chickpea (*Cicer arietinum* L.): a review. Br J Nutr. 2012;108:S11–S26.

9. Harlan JR, Zohary D. Distribution of wild wheats and barley. Science. 1966;153:1074–80.

10. Food and Agriculture Organization of the United Nations. Crops: FAOSTAT statistics database. 2017. <www.fao.org/faostat/en/#data/QC>.

11. Consortium IWGS. Shifting the limits in wheat research and breeding using a fully annotated reference genome. Science. 2018;361(661):1–13.

12. Zeng L, Tu XL, Dai H, Han FM, Lu BS, Wang MS, et al. Whole genomes and transcriptomes reveal adaptation and domestication of pistachio. Genome Biol. 2019;20(1):79.

13. Guo MX, Zhang ZR, Li SP, Lian Q, Fu PC, He YL, et al. Genomic analyses of diverse wild and cultivated accessions provide insights into the evolutionary history of jujube. Plant Biotechnol J. 2021;19(3):517–31.

14. Elfaki MO, Abdelatti KA. Rumen content as animal feed a review. U K J Vet Med Anim Prod. 2016;7:80–88.

15. Zhou TY, Lu LL, Yang G, Al–Shehbaz IA. *Brassica* Linnaeus. In: Wu ZY, Raven PH, Hong DY, editors. Flora of China 8. Beijing and St Louis: Science Press and Missouri Botanical Garden Press; 2000. p. 1–193.

16. McGranahan G, Leslie CA. Walnuts (*Juglans*). In: Moore JN, Ballington JR, editors. Genetic resources of temperate fruit and nut crops. Wageningen: International Society for Horticultural Science; 1991. p. 907–51.

17. Li H, Liu Q, Zhang Q, Qin E, Jin C, Wang Y, et al. Curd development associated gene (CDAG1) in cauliflower (*Brassica oleracea* L. var. *botrytis*) could result in enlarged organ size and increased biomass. Plant Sci. 2017;254:82–94.

18. Anthony RG, James PE, Jordan BR. The cDNA sequence of acauliflower apetala–1/squamosa homolog. Plant Physiol. 1995;108:441–442.

19. Anthony RG, James PE, Jordan BR. Cauliflower (*Brassica oleracea* var. *botrytis*) curd development: the expression of meristem identity genes. J Exp Bot. 1996;47:181–8.

20. Cheung KL, Kong AN. Molecular targets of dietary phenethyl isothiocyanate and sulforaphane for cancer chemoprevention. The AAPS J. 2010;12(1):87–97.

21. Myles S, Boyko AR, Owens CL, Brown PJ, Grassi F, Aradhya MK, et al. Genetic structure and domestication history of the grape. P Nat Acad Sci U.S.A. 2011;108(9):3530–5.

22. Morelock T, Correll J. In: Prohens J, Nuez F. editor. Vegetables I. New York: Springer; 2008. p. 189–218.

23. Che G, Zhang X. Molecular basis of cucumber fruit domestication. Curr Opin Plant Biol. 2019;47:38–46.

24. Zhao J, Jiang L, Che G, Pan Y, Li Y, Hou Y, et al. A functional allele of CsFUL1 regulates fruit length through repressing CsSUP and inhibiting auxin transport in Cucumber. The Plant cell. 2019;31(6):1289–1307.

25. Lu C, Ji D. The cultivars of Mulberry in China. Chongqing: Southwest China Normal University Press; 2017.

26. Wang L. The cultivation evolution and farming culture as well as resources utilization of mulberry. Master's degree: Hongkai University of Agriculture and Engineering; 2019.

27. Liu R, Yang T, Huang YN, Zong XX. Research progress of germplasm resources of pea and its wild relatives. J Plant Genetic Res. 2020;21(6):1415–23.

28. Ma DQ, Xu TW, Gu MZ, Wu SB, Kang YC. The classification and distribution of wild barley in the Tibet Autonomous Region. Scientia Agri Sinica. 1987;20(2):1–6.

29. Ma DQ, Xu TW. The research on classification and origin of cultivated barley in Tibet Autonomous Region. Scientia Agri Sinica. 1988;21(5):7–14.

30. Dai F, Nevo E, Wu D, Comadran J, Zhou M, Qiu L, et al. Tibet is one of the centers of domestication of cultivated barley. P Nati Acad Sci USA. 2012;109(42):16969–73.

31. Ren XF, Nevo E, Sun DF, Sun GL. Tibet as a potential domestication center of cultivated barley of China. PloS One. 2013;8(5):e62700.

32. Zeng X, Guo Y, Xu Q, Mascher M, Guo G, Li S, et al. Origin and evolution of qingke barley in Tibet. Nat Commun. 2018;9(1):5433.

33. Prain D. The mustards cultivated in Bengal. Agr Ledger. 1989;5:1–80.

34. Sinskaia EN. The oleiferous plants and root crops of the family Cruciferae. Bull Appl Bot Genet and pl Breed. 1928;19:641–8.

35. Tan JJ. Origin and taxonomy of *Brassica*. J Hebei Agri Univer. 1980;4(1):111–3.

36. Li JW. The origins and variations of vegetable crops in China. Scientia Agri Sinica. 1981; 1:90–5.

37. Chen SR. The origin and differentiation of mustard varieties in China. Cruciferae Newsl. 1982;7:7–10.

38. Burkill IH. The Chinese mustard in the Malay Peninsula. Gads Bull. 1930;5:99–117.

39. Vaughan JG, Hemingway JS, Schofield HJ. Contributions to a study of variation in *Brassica juncea* Coss. & Czern. J Linn Soc (Bot) 1963;58(374):435–47.

40. Liu SZ, You L, Yang L, Chen H, Yang B, Kang L. Origin and domestication discovery of *Brassica juncea* Cezrn. et Coss. Chinese J Oil Crop Sci. 2018;40(5):649–55.

41. Kang L, Qian L, Zheng M, Chen L, Chen H, Yang L, et al. Genomic insights into the origin, domestication and diversification of *Brassica juncea*. Nat Genet. 2021;53(9):1392–402.

42. Linquvist K. On the origin of cultivated lettuce. Hereditas. 1996;46:319–50.

43. Mou B. Lettuce. In: Prohens J, Nuez F, editors. Handbook of plant breeding, Vol I: Vegetables I: Asteraceae, Brassicaceae, Chenopodicaceae, and Cucurbitaceae. New York: Springer Science; 2008. p. 75–116.

44. Oost E. Domesticatie en verdere ontwikkeling van sla, witlof en andijvie. Wageningen Agricultural University: Ingenieurs Thesis; 1980.

45. Zhang L, Su W, Tao R, Zhang W, Chen J, Wu P, et al. RNA sequencing provides insights into the evolution of lettuce and the regulation of flavonoid biosynthesis. Nat Commun. 2017;8(1):2264.

46. Wei T, van Treuren R, Liu X, Zhang Z, Chen J, Liu Y, et al. Whole–genome resequencing of 445 *Lactuca* accessions reveals the domestication history of cultivated lettuce. Nature Genet. 2021;53(5):752–60.

47. Li QY, Yang MX. Preliminary investigation on buckwheat origin in Yunnan. In: Lin R, Zhou M, Tao Y, Li J, Zhang Z, editors. Proceedings of the 5th International Symposium on Buckwheat, Taiwan, China. Agricultural Publishing House; 1992. p. 44–6.

48. Murai M, Ohnishi O. Population genetics of cultivated common buckwheat, *Fagopyrum esculentum* Moench. X. diffusion routes revealed by RAPD markers. Genes Genet Syst. 1996;71:211–8.

49. Li X, Shang X, Dodson J, Zhou X. Holocene agriculture in the Guanzhong Basin in NW China indicated by pollen and charcoal evidence. The Holocene. 2009;19(8):1213–20.

50. Li XQ, Zhou XY, Zhou J, Dodson J, Zhang HB, Shang X. The earliest agricultural diversification recorded by biological indicators in Gansu Xishanping ruins (In Chinese). Sci Sin. 2007;37(7):934–40.

51. Li M, Yang X, Wang H, Wang Q, Jia X, Ge Q. Starch grains from dental calculus reveal ancient plant foodstuffs at Chenqimogou site, Gansu Province. Sci China Earth Sci. 2010; 53(5):694–9.

52. Jia X. Study on the cultural evolution and vegetable remains of the Neolithic–Bronze Age in Northeastern Qinghai (In Chinese). Doctor Dissertation: Lanzhou University; 2012.

53. Wang Q. Analysis of remain plants in Xueshan ruins in Chengjiang County, Yunnan (In Chinese). Doctor Dissertation: Shandong University; 2014.

54. Sun YG, Zhao ZJ. The flotation results and analysis to the Liao Dynasty Ruins in Bayan Tala Town, Chifeng City, Inner Mongolia. Southern Cul Relics. 2014;3:68–71.

55. Yang C, Xu K, Zhao ZJ. Analytical report on the flotation results of Sunchangqing ruins in Baicheng City, Jilin Province (In Chinese). Northern Cul Relics. 2010;4:48–51.

56. Wei YM. Discovery and chronological analysis of buckwheat kernel in Donghuishan ruins. Crops. 2019;1:85–89.

57. Varshney RK, Thudi M, Roorkiwal M, He W, Upadhyaya HD, Yang W, et al. Resequencing of 429 chickpea accessions from 45 countries provides insights into genome diversity, domestication and agronomic traits. Nature Genet. 2019;51(5):857–64.

58. Zohary D, Hopf M, Weiss E. Domestication of plants in the old world: the origin and spread of domesticated plants in southwest Asia, Europe, and the Mediterranean Basin. Oxford: Oxford University Press; 2012.

59. Petrie CA. Mehgarh, Pakistan. In: Barker G, Goucher C, editors. The Cambridge World History Volume II–World with Agriculture, 12000 BCE–500 CE. Cambridge: Cambridge University Press; 2015. p. 289e309.

60. Liu X, Jones PJ, Motuzaite Matuzeviciute G, Hunt HV, Lister DL, An T, et al. From ecological opportunism to multi–cropping: Mapping food globalisation in prehistory. Quaternary Sci Rev. 2019;206:21–8.

61. Doumani PN, Frachetti MD, Beardmore R, Schmaus TM, Spengler RN, Mar'yashev AN. Burial ritual, agriculture, and craft production among Bronze Age pastoralists at Tasbas (Kazakhstan). Archaeol Res Asia. 2015;1–2:17–32.

62. Fuller DQ. Finding plant domestication in the Indian Subcontinent. Curr Anthropol. 2011; 52(S4):S347–S62.

63. Zhou X, Yu J, Spengler RN, Shen H, Zhao K, Ge J, et al. 5,200–year–old cereal grains from the eastern Altai Mountains redate the trans–Eurasian crop exchange. Nat Plants. 2020; 6(2):78–87.

64. Poets AM, Fang Z, Clegg MT, Morrell PL. Barley landraces are characterized by geographically heterogeneous genomic origins. Genome Biol. 2015;16:173.

65. Mascher M, Schuenemann VJ, Davidovich U, Marom N, Himmelbach A, Hubner S, et al. Genomic analysis of 6,000–year–old cultivated grain illuminates the domestication history of barley. Nature Genet. 2016;48(9):1089–93.

66. Liu MJ, Wang M. Germplasm resources of Chinese jujube. Beijing: China Forestry Publishing House; 2009.

67. Qu Z, Wang Y. Chinese fruit trees record–Chinese jujube. Beijing: China Forestry Publishing House; 1993.

68. Crane JC. Pistachio tree nuts. Westport: Avipublishing Company; 1978.

69. Hormaza JI, Dollo L, Polito VS. Determination of relatedness and geographical movements of *Pistacia vera* (Pistachio; Anacardiaceae) germplasm by RAPD analysis. Econ Bot. 1994; 48:349–58.

70. Hormaza JI, Plnney K, Polito VS. Genetic diversity of Pistachio (*Pistacia vera*, Anacardiaceae) germplasm based on Randomly Amplified Polymorphic DNA (RAPD) markers. Econ Bot. 1998;52:78–87.

71. Sun QZ, Liu Q, Li F, Tao Y. Alfalfa in ancient China: botanical aspects. Acta Prat Sinica 2016;25(5):202–13.

72. Vavilov NI. Phytogeographic basis of plant breeding. Chron Bot. 1951;13:14–56.

73. Griffiths FP. Production and utilization of alfalfa. Econ Bot. 1949;3(2):170–83.

74. Geng H. Alfalfa in China. Beijing: China Agriculture Press; 1995.

75. Shen C, Du H, Chen Z, Lu H, Zhu F, Chen H, et al. The Chromosome–level genome sequence of the autotetraploid alfalfa and resequencing of core germplasms provide genomic resources for alfalfa research. Mol Plant. 2020;13(9):1250–61.

76. del Carpio DP, Basnet RK, de Vos RC, Maliepaard C, Visser R, Bonnema G. The patterns of population differentiation in a *Brassica rapa* core collection. Theor Appl Genet. 2011; 122(6):1105–18.

77. Guo Y, Chen S, Li Z, Cowling WA. Center of origin and centers of diversity in an ancient crop, *Brassica rapa* (Turnip Rape). J Hered. 2014;105(4):555–65.

78. Pang W, Li X, Choi SR, Dhandapani V, Im S, Park MY, et al. Development of a leafy *Brassica rapa* fixed line collection for genetic diversity and population structure analysis. Mol Breeding. 2015;35:54.

79. Zhang CD. Vegetables historical narrative · Turnip. China Veget. 2012;9:43.

80. Qi X, An H, Ragsdale AP, Hall TE, Gutenkunst RN, Chris Pires J, et al. Genomic inferences of domestication events are corroborated by written records in *Brassica rapa*. Mol Ecol. 2017;26(13):3373–88.

81. Roor W, Konrad H, Mamadjanov D, Geburek T. Population differentiation in common walnut (*Juglans regia* L.) across major parts of its native range–insights from molecular and morphometric data. J Hered. 2017;108(4):391–404.

82. Liu Y, Wang C. Origin and classification of *Brassica oleracea* L. Northern Hortic. 2006; 4:58–60.

83. McGovern PE. Ancient wine: the search for the origins of viniculture. Princeton: Princeton University Press; 2003.

84. Olmo H. Grapes. In: Smartt J, Simmonds N, editors. Evolution of crop plants, 2nd edition. New York: Longman; 1995. p. 485–90.

85. Ribera A, van Treuren R, Kik C, Bai Y, Wolters AMA. On the origin and dispersal of cultivated spinach (*Spinacia oleracea* L.). Genet Resour Crop Ev. 2020;68(3):1023–32.

86. Xu C, Jiao C, Sun H, Cai X, Wang X, Ge C, et al. Draft genome of spinach and transcriptome diversity of 120 *Spinacia* accessions. Nat Commun. 2017;8:15275.

87. Cornille A, Giraud T, Smulders MJ, Roldan–Ruiz I, Gladieux P. The domestication and evolutionary ecology of apples. Trends Genet. 2014;30(2):57–65.

88. Sun X, Jiao C, Schwaninger H, Chao CT, Ma Y, Duan N, et al. Phased diploid genome assemblies and pan–genomes provide insights into the genetic history of apple domestication. Nat Genet. 2020;52:1423–32.

89. Sebastian P, Schaefer H, Telford IR, Renner SS. Cucumber (*Cucumis sativus*) and melon (*C. melo*) have numerous wild relatives in Asia and Australia, and the sister species of melon is from Australia. P Nati Acad Sci USA. 2010;107(32):14269–73.

90. Paris HS. Overview of the origins and history of the five major cucurbit crops: issues for ancient DNA analysis of archaeological specimens. Veg Hist Archaeobot. 2016;25(4):405–14.

91. Jiao F, Luo R, Dai X, Liu H, Yu G, Han S, et al. Chromosome–level reference genome and population genomic analysis provide insights into the evolution and improvement of domesticated mulberry (*Morus alba*). Mol Plant. 2020;13(7):1001–12.

92. Zohary D, Hopf M, Weiss E. Domestication of plants in the old world: the origin and spread of domesticated plants in southwest Asia, Europe, and the Mediterranean Basin. Oxford: Oxford University Press; 2012.

93. Riehl S, Zeidi M, Conard NJ. Emergence of agriculture in the foothills of the Zagros Mountains of Iran. Science. 2013;3441(5):65–7.

94. Zheng ZJ, Wang SM, Zong XX. Chinese edible legume. Beijing: China Agriculture Press; 1997.

95. Kreplak J, Madoui MA, Capal P, Novak P, Labadie K, Aubert G, et al. A reference genome for pea provides insight into legume genome evolution. Nat Genet. 2019;51(9):1411–1422.
